# Nutritional management of search and rescue dogs

**DOI:** 10.1017/jns.2017.47

**Published:** 2017-08-29

**Authors:** G. Vassalotti, N. Musco, P. Lombardi, S. Calabrò, R. Tudisco, V. Mastellone, R. Grazioli, S. Bianchi, M. I. Cutrignelli

**Affiliations:** 1 Department of Veterinary Medicine and Animal Production, University of Naples Federico II, Napoli, Italy; 2 Farmina Pet-food, Department of Research and Development, Nola, Napoli, Italy

**Keywords:** Osteo-articular apparatus, *n*-3 Fatty acids, Glucosamine, Search and rescue dogs, Chondroitin sulfate, HS, high supplementation, LS, low supplementation, ME, metabolisable energy, OA, osteoarthritis, SAR, search and rescue

## Abstract

Dogs used for search and rescue (SAR) may experience continuous micro-traumas that predispose them to skeletal disorders. The aim of the present study was to evaluate the effect of diet on osteo-articular apparatus in healthy SAR dogs. A total of sixteen SAR dogs were divided into two groups (low supplementation (LS) and high supplementation (HS)) and were fed for 3 months with two experimental diets, characterised by the same protein and energy density, but different in *n*-3 PUFA (6·2 *v*. 8·4 % of metabolisable energy), chondroitin sulfate (219·8 *v*. 989·0 mg/kg DM) and glucosamine (769·2 *v*. 1318·7 mg/kg DM) in the LS and HS groups, respectively. At recruitment all dogs showed no joint inflammation signs, except four that showed mild symptoms. Haematology and serum biochemistry were performed every 30 d. Joint status was scored by physical and lameness evaluations. The sampling effect analysis showed potential beneficial effects by a decrease in a specific marker of membrane integrity (creatine kinase; CK). Comparing groups, glucose was significantly higher and CK was significantly lower in the HS group; however, in both cases the levels of these parameters fell in the normal range. At the end of the experiment, erythrocytes, Hb and packed cell volume were significantly higher in the HS compared with the LS group. These could result in an improvement in dogs’ performance, even if this aspect was not investigated in the present study. Concerning joint evaluation (pain on manipulation, lameness and range of motion), no statistically significant differences were detected between the groups and within the experimental period.

Bone and joint diseases affect both young (birth defects) and ageing (degenerative arthritic damage) dogs. Osteoarthritis (OA) is a chronic and progressive articular cartilage degradation associated with thickening, sclerosis of subchondral bone, synovitis and fibrosis^(^^1^^)^. An increased incidence of OA has been recently observed due to the increase of obesity in dogs^(^^2^^,^^3^^)^.

OA is a disease caused by genetic and environmental factors that increases cartilage damage promoting degenerative changes. Since it adversely affects the quality of a dog's life, often leading to euthanasia, it has made dog owners much more sensitive to orthopaedic diseases^(^^4^^)^.

As reported by Kivranta *et al*.^(^^5^^)^, vigorous running exercises alter the loading pattern of joints. Since dogs used for search and rescue (SAR) start their training programme at 1 year of age, their osteo-articular apparatus is highly stressed. For this reason, SAR dogs in the rubble show early clinical signs of osteo-articular impairment due to continuous micro-traumas. One of the approaches for preventing joint stress is to include in the diet specific substances capable of both protecting and improving joint function.

Several authors reported beneficial effects of some *n*-3 PUFA, in particular EPA and DHA from fish oil, due to the reduction of inflammatory states^(^^6^^,^^7^^)^ by acting on pro-inflammatory cytokine production^(^^8^^)^ and to the improvement of synovial fluid equilibrium^(^^9^^)^.

Several studies in dogs showed a marked reduction in arthritic pain with the oral administration of glucosamine and chondroitin^(^^10^^,^^11^^)^. Both these chondroprotectants are widely used to treat the pain and loss of function associated with OA. It is believed that glucosamine helps cartilage formation and repair, and that chondroitine improves the elastic properties of cartilage and reduces painful swelling in joints^(^^12^^)^. The oral administration of *n*-3 PUFA and chondroprotectants is usually controversial. In particular, the formulation of the diet as well as the amount of substance to use appear to be critical^(^^13^^)^.

The aim of the present study was to evaluate the effects of a specific supplementation (*n*-3 PUFA, chondroitin sulfate and glucosamine) on the skeletal welfare in dogs used for SAR.

We hypothesised that diets supplemented with high amounts of glucosamine, chondroitin sulfate and *n*-3 PUFA would improve the osteoarthritic condition in SAR dogs.

## Materials and methods

The study was reviewed by the Ethical Animal Care and Use Committee of the University of Naples Federico II and received formal institutional approval in accordance with local and national law, regulations and guidelines.

A total of sixteen healthy dogs used by SAR firefighters (age 5·1 (sd 2·7) years; mean weight 24·7 (sd 6·9) kg; body condition score 4·5 (sd 1·0) out of 9) were selected and equally divided into two groups (low supplementation (LS) and high supplementation (HS)) homogeneous for age, sex and body weight. At recruitment, no animal showed serious signs of joint inflammation, only four dogs (two per group) showed mild symptoms of inflammation, such as pain caused by manipulation or decrease of range of motion and lameness.

In accordance with the habits used for Italian firefighter SAR dogs, all animals were housed with their owners and followed a home daily fitness regimen (1 h) and a weekly training (2 h) including rescuing, obedience and gym provided to fit the testing standards for rescue dogs by the International Rescue Dog Organisation (IRO).

Before the trial the dogs were fed different diets, not specifically formulated for articular management. Consequently, before recruitment each dog was progressively adapted to the experimental diets for 2 weeks.

Dogs were fed for 3 months with two experimental diets having the same energy density (metabolisable energy (ME) 14·40 (sd 0·03) MJ/kg), protein concentration (22·38 (sd 0·15) % ME) and N-free extract level (46·55 (sd 0·09) % ME), but different content of *n*-3 fatty acids, mainly EPA and DHA (6·2 *v*. 8·4 % ME for the LS and HS groups, respectively), chondroitin sulfate (219·8 *v*. 989·0 mg/kg DM, for the LS and HS groups, respectively) and glucosamine (769·2 *v*. 1318·7 mg/kg DM, for the LS and HS groups, respectively). Diet ingredients and chemical composition are reported in Supplementary Table S1. Chondroitin sulfate from pigs (concentration 90 %), glucosamine from aquatic animals (concentration 95 %) and fish oil (source of EPA and DHA) were purchased from Mazzoleni Spa.

During the first visit the nutritional requirements according to live weight, body condition score and training activity were calculated for each dog. None of the dogs had received any therapeutic treatment for at least 3 months before the onset of the experiment.

Dogs were clinically evaluated, before and after X-rays were taken, and the dogs underwent blood collection every 30 d for 3 months. In order to perform haematology and clinical chemistry analysis blood was taken in fasted dogs at 08.00 hours from the jugular vein into plastic tubes with and without K3-EDTA. Serum was obtained by centrifugation at 1500 ***g*** for 15 min.

A complete blood count was performed by a Lasercyte Haematology Analyser (Idexx Laboratories). Blood urea N, creatinine, glucose, cholesterol, TAG, alanine amino transferase, aspartate amino transferase, γ-glutamyl transferase, alkaline phosphatase, bilirubin, total protein, albumin, lactic dehydrogenase and creatine kinase (CK) were measured by an AMS Autolab spectrophotometer using reagents from Spinreact before and after 30, 60 and 90 d of diet administration. Joint status was scored 0 (normal), 1 (mild), 2 (moderate) and 3 (severe) as proposed by Pollmeier *et al*.^(^^14^^)^ by physical and lameness evaluations (lameness, pain on manipulation and palpation, range of motion and joint swelling). All evaluations were performed by the same veterinary practitioner, who was blinded to the treatment groups.

The effects of sampling times and between groups were analysed by ANOVA using the Proc GLM of SAS^(^^15^^)^ according to the following model:
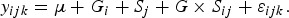
where *y* is the dependent variable, *μ* is the mean, *G* is the group effect (*i* = LS, HS), *S* is the sampling effect (*j* = 0, 30, 60, 90), *G* × *S* is the first level of interaction and *ε* is the error effect. When significant differences were found in the ANOVA, means were compared using Tukey's test. The clinical signs scores were statistically evaluated using the Wilcoxon non-parametric test (PROC NPAR1WAY of SAS)^(^^15^^)^.

## Results

Weight loss (−3·9 and −3·7 %) and a body condition score decrease (from 5 to 4 out of 9) were observed in both groups, starting from day 30, though the differences were not significant (*P* > 0·05) (data not shown).

All biochemistry and haematology parameters fall within the normal range for both groups. For brevity, only a few parameters of haematology are reported in Table 1 whereas for serum biochemistry, bilirubin, total protein and albumin were omitted. At the end of the experiment, erythrocytes, Hb and packed cell volume were significantly higher in the HS group compared with the LS group (*P* < 0·01 and *P* < 0·05). Glucose was significantly (*P* < 0·01) higher and CK was significantly (*P* < 0·01) lower in dogs of the HS group compared with the LS group. As shown in Table 1, no statistical difference between groups was recorded for the other parameters.

**Table 1.** Main results of complete blood count and biochemistry parameters (*n* 16)

	Blood count			Biochemistry										
	Erythrocytes (cells/μl)	Hb (g/dl)*	Packed cell volume (%)	BUN (mg/dl)*	Creatinine (mg/dl)*	AST (U/l)	ALT (U/l)	GGT (U/l)	ALP (U/l)	Glucose (mg/dl)†	Cholesterol (mg/dl)‡	TAG (mg/dl)§	LDH (U/l)	CK (U/l)
Group effect														
LS	6·67	15·41	43·84	25·78	0·894	37·87	52·81	6·25	148·09	82·03	283·00	74·00	194·44	65·84
HS	7·28	16·43	46·71	24·41	0·900	36·12	54·07	5·97	149·61	93·46	299·78	70·62	165·14	53·34
*P*	0·0024	0·0005	0·0316	0·5058	0·9171	0·4940	0·7369	0·7198	0·9216	0·0014	0·3421	0·6496	0·2652	0·0006
Sampling effect (d)														
0	6·72	15·38	42·75	29·42	1·000	36·87	60·39	8·80	114·60	92·46	380·03	64·69	198·28	96·51
30	6·90	16·18	44·18	20·05	0·545	50·38	57·41	5·79	163·57	88·02	213·37	83·47	152·45	55·55
60	7·12	16·35	46·47	25·31	0·939	28·73	51·87	5·78	159·93	84·43	311·69	74·12	217·77	45·47
90	7·17	15·78	47·71	25·60	1·100	32·01	44·10	4·07	157·30	86·08	260·47	66·96	150·65	40·83
*P*	0·3162	0·072	0·0408	0·0208	<0·0001	<0·0001	0·0174	0·0007	0·0926	0·3810	<0·0001	0·2807	0·1857	<0·0001
Group × sampling *P*	0·9768	0·575	0·7093	0·6361	0·1466	0·1936	0·6905	0·3817	0·6649	0·0021	0·2558	0·8273	0·1344	0·0321
RMSE	0·735	1·071	5·016	7·904	0·231	9·857	14·401	2·947	59·104	13·124	67·648	28·543	100·51	13·134

BUN, blood urea N; AST, aspartate amino transferase; ALT, alanine amino transferase; GGT, γ-glutamyl transferase; ALP, alkaline phosphatase; LDH, lactic dehydrogenase; CK, creatine kinase; LS, low supplementation (EPA + DHA: 6·2 % metabolisable energy; chondroitin sulfate: 219·8 mg/kg DM; glucosamine: 769·2 mg/kg DM); HS, high supplementation (EPA + DHA: 8·4 % metabolisable energy; chondroitin sulfate: 989·0 mg/kg DM; glucosamine: 1318·7 mg/kg DM); RMSE, root mean square error.

* To convert mg/dl or g/dl to mg/l or g/l, multiply by 10.

† To convert glucose in mg/dl to mmol/l, multiply by 0·0555.

‡ To convert cholesterol in mg/dl to mmol/l, multiply by 0·0259.

§ To convert TAG in mg/dl to mmol/l, multiply by 0·0113.

Regarding the sampling time, the statistical analysis conducted showed a significant reduction in creatinine, aspartate amino transferase, γ-glutamyl transferase, cholesterol and CK (*P* < 0·01), as well as blood urea N and alanine amino transferase (*P* < 0·05). Considering the significant group × sampling interaction observed for glucose and CK, a particular attention was paid to the trend during the trial of these parameters for each group. Regarding glucose level, the LS group showed a slight increase from the first to the last sampling, which was not statistically significant (*P* > 0·05); on the contrary in the HS group, which showed a higher baseline value than the LS group (mean value 5·55 (sd 0·50) *v.* 4·22 (sd 0·78) mmol/l (100 (sd 9) *v*. 76 (sd 14) mg/dl), respectively), a progressive and significant (*P* < 0·05) decrease was observed. Both groups showed a significant (*P* < 0·0001) decline in CK all through the experiment; between groups, the HS group showed significantly (*P* < 0·0006) lower CK compared with the LS group.

**Fig. 1.**
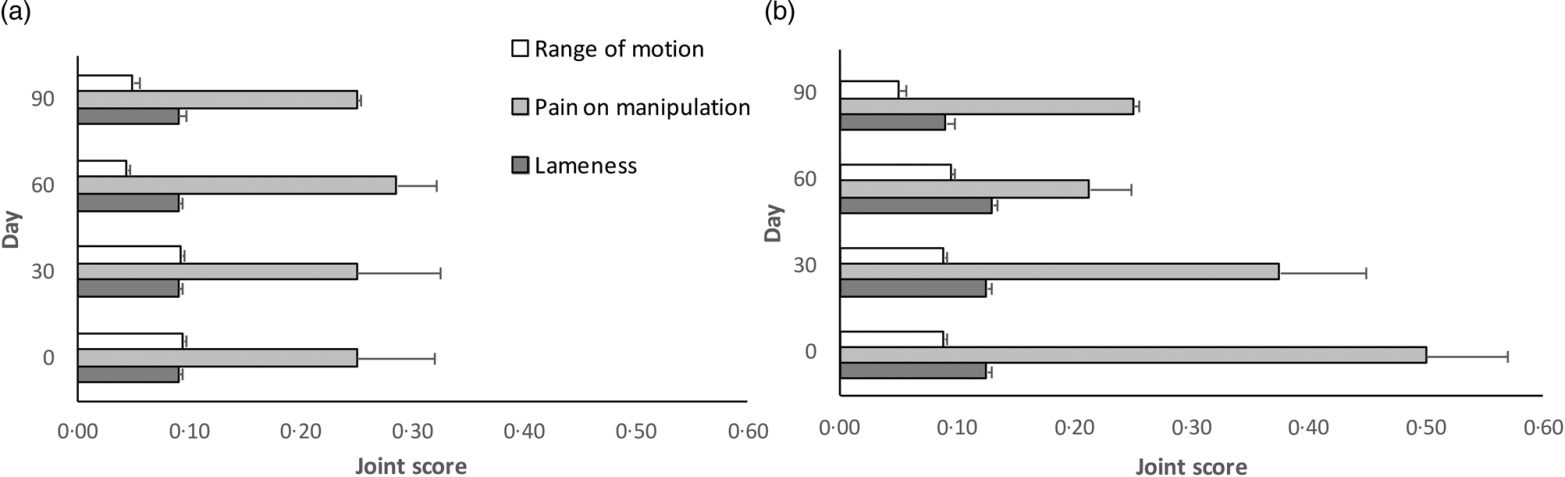
Joint evaluation during the trial (*n* 16). (a) Low supplementation group (EPA + DHA: 6·2 % metabolisable energy; chondroitin sulfate: 219·8 mg/kg DM; glucosamine: 769·2 mg/kg DM); (b) high supplementation group (EPA + DHA: 8·4 % metabolisable energy; chondroitin sulfate: 989·0 mg/kg DM; glucosamine: 1318·7 mg/kg DM). Joint score: 0 (normal), 1 (mild), 2 (moderate), 3 (severe). Values are means, with standard deviations represented by horizontal bars. Group effects *P* > 0·714, *P* > 0·949 and *P* > 0·709 for range of motion, pain of manipulation and lameness, respectively. Sampling effects *P* > 0·357, *P* > 0·491 and *P* > 0·354 for range of motion, pain of manipulation and lameness, respectively.

In both groups, joint status (Fig. 1) was always below the score of 1 for all parameters; no dog was found positive for joint swelling.

No statistically significant (*P* > 0·05) differences were detected for the non-parametric data (range of motion, pain of manipulation and lameness) considering both group (*P* > 0·7) and sampling effects (*P* > 0·3) along the experimental period. Indeed, a decrease of the joint score was recorded at day 90 for pain on manipulation, lameness and range of motion in the HS group.

X-ray images of the joints before and after the nutritional treatment did not show any sign of osteo-articular injury.

## Discussion and conclusions

All parameters fall within the normal range, thus showing, together with the absence of clinical signs, that the nutritional supplement had no adverse effect^(^^16^^)^.

The diet supplementation with *n*-3 fatty acids, chondroitin sulfate and glucosamine showed effects on blood count parameters. The difference between groups gave contrasting results. The amount of *n*-3 PUFA, chondroitin sulfate and glucosamine contained in the two diets did not seem to be critical in determining their effects. Dogs from the HS group showed higher values of erythrocytes, Hb and packed cell volume; this may suggest an increased physical efficiency. Such kinds of haematological changes may result in increasing energy production during exercise^(^^16^^)^ and, potentially, in improving dog performance. Since all dogs underwent the same training programme all through the trial, the possible benefits should need a specific evaluation of the athletic performances. CK is considered to be a marker of muscle function; therefore, falling in the physiological range, its reduction may be suggestive of an improvement in muscular membrane integrity. Such effect could be due to the antioxidant properties of *n*-3 fatty acids that, by contrasting the effects of reactive oxygen species, are able to improve cell integrity^(^^17^^)^. On the other hand, since no difference was observed for the other markers of muscle wellness (aspartate amino transferase and lactic dehydrogenase), these results need to be investigated in depth.

Glucosamine and chondroitin, having been reported to improve joint function^(^^13^^)^, may be responsible for the improvement in pain on manipulation, lameness and range of motion. Indeed, such improvement could be also due to the antioxidant effect of *n*-3 PUFA by improving the articular synovia^(^^9^^)^. According to Beale^(^^13^^)^, it is difficult to identify the exact nutraceutical treatment that leads to joint improvement. In any event, this study shows that the combination of chondroitin sulfate, glucosamine and *n*-3 PUFA in the diet improves joint status. The possible effects of such improvement on dogs’ athletic performance as well as on their athletic life need further study.

## Supplementary material

For supplementary material accompanying this paper visit http://dx.doi.org/10.1017/jns.2017.47. click here to view supplementary material
