# Supplementary material for: Nutritional management of search and rescue dogs
Source: J Nutr Sci. 2017 Aug 29;6:e44. doi: 10.1017/jns.2017.47 (PMC5672312; doi:10.1017/jns.2017.47)
Supplement: Supplementary file 1 [file S2048679017000477sup001.doc]

**Supplementary Table S1.** Ingredients and chemical composition of the diets

| Ingredients: Spelt, oats, dehydrated chicken meat, linseed, animal fat, dehydrated whole eggs, dried beet pulp, hydrolysed animal proteins, fish oil, pea fibre, potassium chloride, fructooligosaccharides, mannanoligosaccharides, dried brewer’s yeast, sodium chloride, glucosamine, chondroitin sulfate | | | |
| --- | --- | --- | --- |
|  |  | LS | HS |
| Dry matter | % | 90.9 | 90.8 |
| MJ/kg DM |  | 15.51 | 15.87 |
| Crude protein | % ME | 22.49 | 22.28 |
| Ether extract | % ME | 29.41 | 29.89 |
| n-3 | % ME | 3.71 | 9.39 |
| n-6 | % ME | 7.66 | 8.40 |
| AA | % ME | 4.26 | 8.28 |
| EPA | % ME | 2.84 | 3.71 |
| DHA | % ME | 3.40 | 4.69 |
| EI | % ME | 46.61 | 46.48 |
| Chondroitine sulphate | mg/kg DM | 181.8 | 817.2 |
| Glucosamine | mg/kg DM | 636.3 | 108.6 |
